# Occurrence of Mycotoxins in Winter Rye Varieties Cultivated in Poland (2017–2019)

**DOI:** 10.3390/toxins12060423

**Published:** 2020-06-26

**Authors:** Robert Kosicki, Magdalena Twarużek, Paweł Dopierała, Bartosz Rudzki, Jan Grajewski

**Affiliations:** 1 Kazimierz Wielki University, Faculty of Biological Sciences, Department of Physiology and Toxicology, Chodkiewicza 30, 85-064 Bydgoszcz, Poland; twarmag@ukw.edu.pl (M.T.); jangra@ukw.edu.pl (J.G.); 2 KWS Lochow Polska Sp. z o. o., Słowiańska 5, Kondratowice, 57-150 Prusy, Poland; pawel.dopierala@kws.com (P.D.); bartosz.rudzki@kws.com (B.R.)

**Keywords:** winter rye, *Fusarium* mycotoxins, trichothecenes, ochratoxin A, chromatography, LC-MS

## Abstract

Rye (*Secale cereale L*.) is one of the most important cereals and is used in both the food and feed industries. It is produced mainly in a belt extending from Russia through Poland to Germany. Despite the great economic importance of this cereal, there is little research on rye contamination with mycotoxins. In this study, the occurrence of *Fusarium* mycotoxins (deoxynivalenol, nivalenol, 3-acetyl-deoxynivalenol, monoacetoxyscirpenol, diacetoxyscirpenol, T-2 toxin, HT-2 toxin, and zearalenone), as well as ochratoxin A, in 60 winter rye samples of four varieties (KWS Binntto, KWS Serafino, Dańkowskie Granat and Farm Saved Seed) cultivated in three consecutive growing seasons in five different regions of Poland was determined using liquid chromatography with tandem mass spectrometry and fluorescence detection. Deoxynivalenol, T-2 toxin, HT-2 toxin, and zearalenone had the highest occurrence in samples (90%, 63%, 57%, and 45% positive results, respectively). The mean concentrations of these analytes were 28.8 µg/kg (maximum 354.1 µg/kg), 0.98 µg/kg (maximum 6.63 µg/kg), 2.98 µg/kg (maximum 29.8 µg/kg), and 0.69 µg/kg (maximum 10.2 µg/kg), respectively. The mean concentrations for individual mycotoxins were highest in the 2016/2017 growing season. In the 2016/2017 growing season, at least two mycotoxins were detected in 95% of the samples, while in the 2018/2019 growing season, 70% of samples contained one or no mycotoxins. The frequencies of mycotoxin occurrence in different rye varieties were similar. Although a high frequency of mycotoxin occurrence was noted (especially deoxynivalenol), their concentrations were low, and none of the analyzed rye samples exceeded the maximum acceptable mycotoxin level set by the European Commission.

## 1. Introduction

Rye (*Secale cereale L.*) is one of the younger cultivated plants. It reached Europe from Central Asia, where even now the wild forms of this plant still grow. Initially, as a weed of cereal crops, it spread to areas of Central and Northern Europe, where plants with higher climatic and soil requirements, such as wheat or barley, subsequently began to be displaced as the conditions of cultivation deteriorated. Rye cultivation began in Europe around 1000 B.C., but it arrived later to Poland, probably in the 5th century B.C. [1].

Although the rye acreage has decreased in recent years, the cool temperate zones of Europe still remain the major rye growing areas. The main producers are Germany (2737 thousand tons), Poland (2673 thousand tons), Russia (2547 thousand tons), Denmark (723 thousand tons), and Belarus (670 thousand tons). In Poland, rye is grown on approximately 873 thousand ha, which accounts for over 8% of all cereals sown. On average, around 2673 thousand tons of grain are obtained in Poland during the year, which constitutes 19.5% of the world harvest [2].

Rye is a typical feed plant. It is given to animals as grain (over 50% of the grain harvest is fed), as well as forage or rye bran. Rye grain is also used in the cereal and milling industry as a raw material for the production of flour (light or dark), from which various types of bread are made. Rye grains contain relatively large amounts of dietary fiber, which includes health-promoting pentosans, fructans, and β-glucans, as well as easily digestible proteins and vitamins. Rye grain also contains phenolic acids and phytoestrogens [3]. Eating rye becomes dangerous when the rye grains contain mycotoxins—secondary metabolites of mold fungi. Mycotoxins can be formed both during plant growth in the field and during grain storage [4]. Field toxins include the ergot alkaloids produced by fungi of the genus *Claviceps* (such as *Claviceps purpurea*), as well as trichothecenes and zearalenone produced by fungi of the *Fusarium* genus (*F. avenaceum*, *F. culmorum*, *F. graminearum*, and *F. nivale*). The mycotoxins produced during storage include ochratoxin A produced by the fungi *Aspergillus ochraceus* and *Penicillium verrucosum*. The production of mycotoxins is closely related to environmental factors, which include: substrate type and consistency, presence of microelements, occurrence of accompanying microflora, humidity, and temperature, of which the latter two are of greatest importance [5]. Gaikpa et al. [6] found that rye is the most resistant grain to *Fusarium* head blight (FHB) and has the lowest kernel damage, compared to triticale, durum wheat and bread wheat.

Trichothecenes are a group of over 150 compounds, among which the most important is group B containing such toxins as nivalenol (NIV) and deoxynivalenol (DON), as well as group A, to which toxins T-2 and HT-2 belong. In Central Europe, deoxynivalenol is one of the most common mycotoxins in cereals and cereal products. It inhibits protein synthesis and gives rise to immunological weakness, while its symptoms are mainly vomiting and decreased appetite. The T-2 and HT-2 toxins have cytotoxic and immunosuppressive effects. Zearalenone (ZEN), due to its structure, has the ability to bind to estrogen receptors in mammals, which can lead to hormonal changes. Ochratoxin A (OTA) is mainly nephrotoxic, but it also has hepatotoxic, teratogenic, mutagenic, and carcinogenic effects [7]. Due to the chemical stability of mycotoxins and the inability to completely eliminate them from raw materials and cereal products, the best way to ensure the safety of humans and animals against the undesirable effects of mycotoxins is to introduce standards regarding their permitted concentration and to systematically monitor their contamination of raw materials and products. In the European Union, maximum levels in rye are set as follows: ochratoxin A, 5 µg/kg (unprocessed rye), 3 µg/kg (all products derived from unprocessed rye); deoxynivalenol, 1.250 µg/kg (unprocessed rye), 750 µg/kg (rye intended for direct human consumption, e.g., rye flour, bran), 500 µg/kg (bread, rye snacks, and breakfast cereals); sum of T-2 and HT-2 toxins, 100 µg/kg (rye), 50 µg/kg (rye for direct human consumption), 100 µg/kg (rye bran), 50 µg/kg (other rye milling products), 25 µg/kg (bread, snacks, and pasta); zearalenone, 100 µg/kg (unprocessed rye), 75 µg/kg (rye intended for direct human consumption, e.g., rye flour, bran), 50 µg/kg (bread, rye snacks, and breakfast cereals) [8,9]. In addition, the European Union has set guidance levels for rye mycotoxin contamination for feed purposes: ochratoxin A, 250 µg/kg (rye and rye products); deoxynivalenol, 8.000 µg/kg (rye and rye products); sum of T-2 and HT-2 toxins, 500 µg/kg (rye and rye products); zearalenone, 2.000 µg/kg (rye and rye products) [10].

The aim of this study was to evaluate the natural occurrence of *Fusarium* mycotoxins and ochratoxin A in winter rye varieties grown in five locations across Poland over a three-year period. Another aspect of the research concerns the co-occurrence of the determined mycotoxins, as well as potential correlations between them.

## 2. Results and Discussion

### 2.1. Weather Data and Agronomic Practise

**Figure 1:**
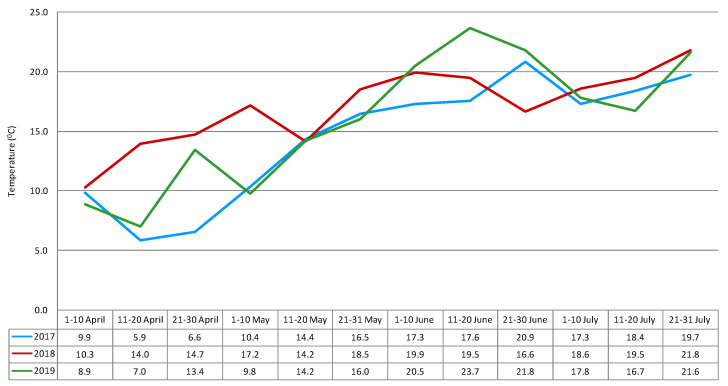
Temperature (ten-days mean) during the growing seasons of winter rye (recorded at Boguszyn).

**Figure 2:**
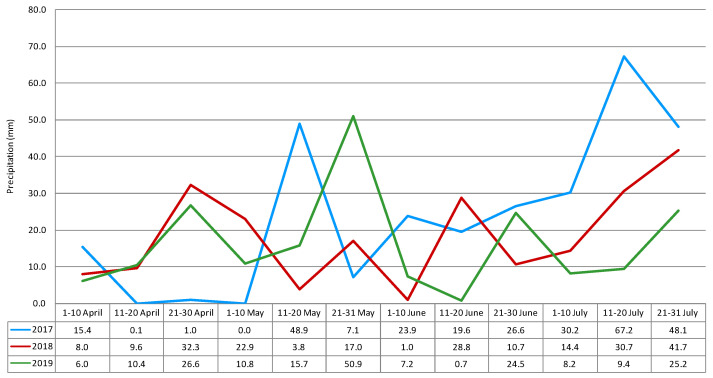
Precipitation (ten-days mean) during the growing seasons of winter rye (recorded at Boguszyn).

Weather conditions (temperature and precipitation level) during the growing season are considered to be the main factors affecting fungal infection and mycotoxin production. Weather conditions in May (flowering) and July (harvest) are crucial for the biosynthesis of mycotoxins in cereals [11]. Exposure to lower temperatures is often connected with higher humidity (higher water activity) and late harvest, and may lead to higher deoxynivalenol concentrations [12,13]. In the 2016/2017 growing season, weather conditions were favorable for the formation of *Fusarium* head blight. From the middle of heading (the 11th to 20th of May) to the end of the early dough stage (the first ten days in July), the rainfall total reached 156.3 mm, and the mean temperature was 17.3 °C (Figure 1 and Figure 2).

These conditions were more favorable for the development of *Fusarium* fungi and rye contamination by mycotoxins compared to the 2017/2018 and 2018/2019 growing seasons, when the total rainfall was 75.7 mm and 107.2 mm, respectively, and the mean temperatures for these periods were 17.9 °C and 19.0 °C, respectively.

In addition to climatic conditions, agricultural practice may have a significant impact on the development of *Fusarium* fungi. For now, however, the results of research on this topic are ambiguous. In Germany, Klix et al. [14] conducted a three-year monitoring of wheat and concluded that tillage and previous crops did not influence the FHB causing species composition in wheat heads. In contrast, in Sweden, Karlsson et al. [15] found that the agricultural practice (application of fertilizers, pesticides and tillage), did have an impact on the amount of some *Fusarium* species in wheat grains. Replacement of conventional farming with organic ones can lead to a reduction in the number of *Fusarium* fungi, and thus a potential reduction in the concentrations of mycotoxins present in cereals. In Norway, Bernhoft et al. [13] revealed that lack of crop rotation, lodged fields, use of mineral fertilizers and, to some extent, use of pesticides (commonly used in conventional farming) were connected to an increase of total *Fusarium* molds.

### 2.2. Occurrence of Mycotoxins

DON is the most prevalent and important mycotoxin occurring in cereal grain in countries with a temperate climate. It belongs to the type B trichothecene group and is produced mainly by *F. graminearum* and *F. culmorum* [16]. In this study, DON was the most common mycotoxin, being detected in 54 samples (90%). No sample exceeded the maximum levels set by the EU [8,10]. All the samples taken in the 2016/2017 growing season contained DON (mean 70.6 µg/kg; maximum 354.1 µg/kg), while in the 2017/2018 and 2018/2019 growing seasons the percentage of positive samples was 90% and 80%, respectively (mean 9.62 µg/kg and 6.21 µg/kg, respectively). Statistically significant differences were found for DON between all three growing seasons (α = 0.05) (Table 1).

As observed in Table 2, the highest mean concentration was determined in Wyczechy (54.1 µg/kg, 100% positive samples), whereas the lowest was in Boguszyn (13.3 µg/kg, 75% positive samples). No statistically significant differences for DON mean concentration were noted only in Boguszyn and Marianowo.

In the presented study (Table 3), the highest mean concentration (43.5 µg/kg) of DON was found in the population of rye from farm reproduction (FSS), while the lowest was in the Dańkowskie Granat rye variety (19.3 µg/kg). Statistically significant differences appeared among all four rye varieties (α = 0.05).

In our previous study, Błajet-Kosicka et al. [17], we analyzed 76 rye grain samples (collected during the 2009–2012 period), DON being detected in 38 of these (50%). Its mean and maximum concentrations were 22.0 µg/kg and 254.0 µg/kg, respectively. In Lithuania, Mankevičienė et al. [18] found that 50% of rye samples were contaminated by DON, with a maximum concentration of 158.9 µg/kg. In one out of 63 rye samples (2%), DON was found at a concentration of 60 µg/kg in a 6-year survey in the Russian Federation [19]. In another study, Remža et al. [20] analyzed 39 Slovak rye grains, and DON was found at mean and maximum concentrations of 67.9 µg/kg and 289.0 µg/kg, respectively. In an earlier study in Germany, Gottschalk et al. [21] detected DON in 100% out of 61 analyzed rye samples, with mean and maximum concentrations of 28.0 µg/kg and 288 µg/kg, respectively. Pleadin et al. [22] analyzed 16 rye samples in Croatia, and DON was found in six of the samples (38%) at a mean concentration of 50.0 µg/kg. In our study, the contamination rate of rye samples was similar to the results reported by Gottschalk et al. [21] and much higher than the results presented by Błajet-Kosicka et al. [17], Mankevičienė et al. [18], Tutelyan et al. [19], Remža et al. [20], and Pleadin et al. [22]. The mean concentration of DON in rye samples was similar to the results obtained by Błajet-Kosicka et al. [17] and Gottschalk et al. [21], and lower than the results reported by Remža et al. [20] and Pleadin et al. [22].

Nivalenol was found in six out of 60 rye samples (10%), giving an overall mean concentration below its detection limit. Its highest concentration (26.4 µg/kg) was determined in Walewice in the 2017/2018 growing season for the KWS Binntto hybrid rye variety. Błajet-Kosicka et al. [17] reported that in two out of 76 rye samples (3%), NIV was detected, with a maximum concentration below the limit of quantification (20 µg/kg). In Germany, NIV was found in 3% of rye samples, and its mean concentration was 0.06 µg/kg [21]. In the presented study, the mean concentration of NIV in the rye samples was similar to the results reported by other researchers [21].

Similarly to nivalenol, low levels of 3-acetyl-deoxynivalenol (3ADON) were determined in the rye samples. The 3ADON was detected in two samples, with the highest concentration being below the limit of quantification (3 µg/kg). The results presented in this study are similar to those obtained earlier in Poland by Błajet-Kosicka et al. [17], who detected 3ADON in two out of seventy-six samples, at a maximum level of <15 µg/kg. Other results were reported by Gottschalk et al. [21]: the maximum concentration was low (5.0 µg/kg), but 3ADON was found in 59% of the samples.

T-2 toxin is the most important type A trichothecene mycotoxin and is produced mainly by *F. sporotrichioides, F. poae,* and *F. equiseti* [4]. T-2 toxin occurred in 63% of the rye samples, and its highest concentration was 6.63 µg/kg. The highest mean concentration of this toxin was recorded in the 2016/2017 growing season (1.64 µg/kg; 80% positive samples), while the lowest was in the 2018/2019 growing season (mean below the limit of quantification; 35% positive samples). The highest mean concentration was determined in Marianowo (2.03 µg/kg, 92% positive samples), while the mean concentrations in Boguszyn and Prusim were below the limit of quantification. In this study, T-2 toxin at the highest mean concentration (1.21 µg/kg) was found in the population of rye from farm reproduction (FSS), while the lowest was in KWS Serafino hybrid rye (0.80 µg/kg). Statistically significant differences were noted between all growing seasons (α = 0.05), as well as between all rye varieties (α = 0.05). In the earlier study in Poland, Błajet-Kosicka et al. [17] found that 30% of 76 rye samples were contaminated with T-2 toxin at a mean content <2 µg/kg (maximum 24.8 µg/kg). In another study with eight rye samples, T-2 toxin was found in 88% of the samples with a maximum value of 20.2 µg/kg [18]. In Russia, Tutelyan et al. [19] analyzed 33 rye samples, and T-2 toxin was found in 12% of the samples with a maximum concentration of 12.0 µg/kg (mean <2 µg/kg). Gottschalk et al. [21] reported that T-2 toxin was detected in 53 (87%) out of 61 rye samples in Germany, at mean and maximum concentrations of 0.13 µg/kg and 0.77 µg/kg, respectively. In our study the contamination rate was lower than that reported by some other authors [18,21] but higher than that reported by Błajet-Kosicka et al. [17] and Tutelyan et al. [19].

HT-2 toxin was detected in 34 samples (57%), and its mean and maximum concentrations were 2.98 µg/kg and 29.8 µg/kg, respectively. The highest mean concentration was obtained in the 2016/2017 growing season (6.59 µg/kg; 85% positive samples). Statistically significant differences for this toxin were found in 2016/2017 growing season (α = 0.05). Similarly to T-2 toxin, the highest mean content of HT-2 toxin was found in Marianowo (7.43 µg/kg, 92% positive samples). No statistically significant differences for HT-2 mean concentration values were observed between Boguszyn, Prusim and Wyczechy. The most contaminated rye varieties were KWS Binntto and rye from farm reproduction (FSS), with mean concentrations of 4.01 µg/kg and 4.04 µg/kg, respectively. The mean content of this mycotoxin was the lowest in the Dańkowskie Granat variety (below the limit of quantification). For HT-2 toxin no statistically significant differences were noted between KWS Binntto and FSS rye varieties. In the earlier survey of rye in Poland, Błajet-Kosicka et al. [17] detected HT-2 toxin in 29% of the samples, with a maximum concentration of 44.1 µg/kg. In five out of 33 rye samples, HT-2 was found at a mean content of <5 µg/kg (maximum 38 µg/kg). In Germany, Gottschalk et al. [21] analyzed 61 rye samples, and HT-2 toxin was found in 57 (93%) samples at mean and maximum concentrations of 0.56 µg/kg and 2.6 µg/kg, respectively. In our study, the mean and maximum levels were similar to the results obtained by Błajet-Kosicka et al. [17], and they were higher than the results reported by Gottschalk et al. [21].

Monoacetoxyscirpenol (MAS) and diacetoxyscirpenol (DAS) are two more mycotoxins belonging to the type A trichothecene group. MAS was detected in 7% of the samples (mean below LOD; maximum 3.07 µg/kg), while DAS was not detected in any rye sample. The highest concentrations of MAS were recorded in the 2017/2018 growing season (mean from positive results 2.29 µg/kg). Similar results were presented by Błajet-Kosicka et al. [17]. MAS was detected in five (7%) out of seventy-six samples with mean and maximum concentrations below the limits of detection (1 µg/kg) and quantification (3 µg/kg), respectively. DAS was not detected in any of the analyzed samples. Gottschalk et al. [21] reported that in 53 (87%) out of 61 rye samples, the mean and maximum contents of MAS were 0.05 µg/kg and 0.31 µg/kg, respectively, whereas DAS was not found in any sample.

Zearalenone is one of the most common *Fusarium* mycotoxins in the temperate climate of Central Europe, being produced mainly by *F. graminearum* and *F. culmorum* [4]. In the presented study, ZEN was detected in 27 samples (45%). In all the samples, ZEN concentrations were below the maximum levels set by the EU [8,10]. Eighty percent of the samples in the 2016/2017 growing symptoms contained ZEN (mean 1.82 µg/kg; maximum 10.2 µg/kg), while in the 2018/2019 growing season the percentage of positive samples was only 5% (mean < LOD, maximum < LOQ). The highest mean concentration was found in Wyczechy (1.80 µg/kg, 42% positive samples), whereas the lowest was in Marianowo (<LOQ, 33% positive samples). In this study, the highest mean concentration (1.13 µg/kg) of ZEN was found in the KWS Binntto hybrid rye, while the lowest was in the KWS Serafino hybrid rye variety (0.35 µg/kg). Statistically significant differences for ZEN mean concentration were determined between all growing seasons (α = 0.05), sample collection locations (α = 0.05), as well as all rye varieties (α = 0.05) (Table 1, Table 2 and Table 3). In our earlier study, Błajet-Kosicka et al. [17], we analyzed 76 rye samples and ZEN was detected in 54% of the samples with mean and maximum concentrations of 4.5 µg/kg and 148 µg/kg, respectively. In Lithuania, Mankevičienė et al. [18] analyzed eight winter rye samples, and ZEN was found in 50% of the samples with a maximum content of 21.2 µg/kg. In another study, Pleadin et al. [22] determined ZEN in 25% of Croatian rye grain samples with mean and maximum contents of 1.44 µg/kg and 7.81 µg/kg, respectively. In the presented study, the level of ZEN rye contamination was similar to the results reported by Błajet-Kosicka et al. [17] and Mankevičienė et al. [18], while higher than the results reported by Pleadin et al. [22]. The maximum ZEN concentration was lower than the results presented by Błajet-Kosicka et al. [17] and Mankevičienė et al. [18], and similar to the results of Pleadin et al. [22].

Ochratoxin A is produced primarily by *Aspergillus ochraceus* and *Penicillium verrucosum* in storage conditions that favor fungal growth and toxin production. There is little information about the conditions needed to produce OTA from fungi in grains during development in the field [4]. No sample exceeded the maximum levels of OTA set by the EU [8,10]. Ochratoxin A was found in two (3%) out of 60 samples of rye grain. Its highest concentration was determined in KWS Serafino hybrid rye in Prusim in the 2016/2017 growing season. In an earlier study, Krysińska-Traczyk et al. [23] analyzed five rye samples in Poland, and OTA was found in 40% of the samples. Its mean and maximum concentrations were 0.19 µg/kg and 0.55 µg/kg, respectively. In another study in Poland, Hajok et al. [24] analyzed 12 samples of rye flour, and OTA was detected in 67% of the samples, with mean and maximum concentrations of 4.3 µg/kg and 19.5 µg/kg, respectively. In Croatia, Pleadin et al. [22] analyzed 16 rye samples and obtained mean and maximum concentrations of 0.89 µg/kg and 4.12 µg/kg, respectively. In 75% of eight rye samples from Serbia, OTA was found at a mean concentration of 3.93 µg/kg (maximum 23.04 µg/kg) [25]. In another study, Cardoso et al. [26] did not find OTA in Portuguese rye flour. In our study, the contamination levels of OTA in rye samples were lower than the results reported by Pleadin et al. [22], Krysińska-Traczyk et al. [23], Hajok et al. [24], and Torović [25].

### 2.3. Co-Occurrence and Correlation Between Mycotoxins

**Figure 3:**
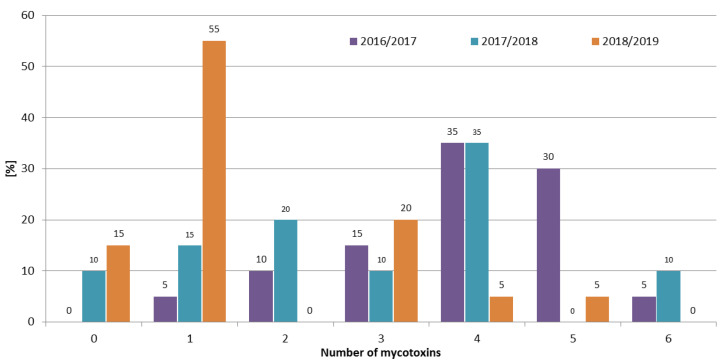
Distribution of the number of mycotoxins in different growing seasons.

**Figure 4:**
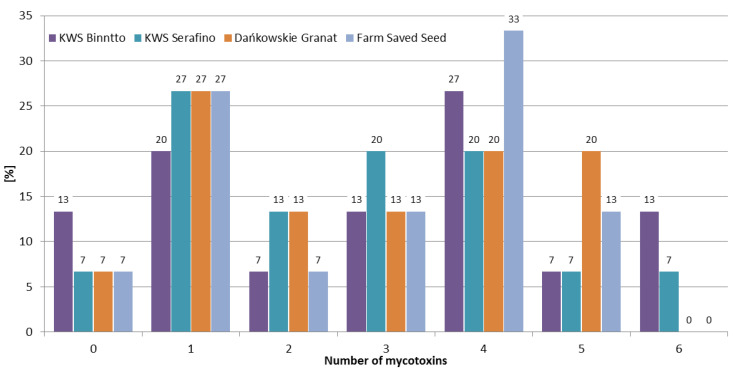
Distribution of the number of mycotoxins in different rye varieties.

**Figure 5:**
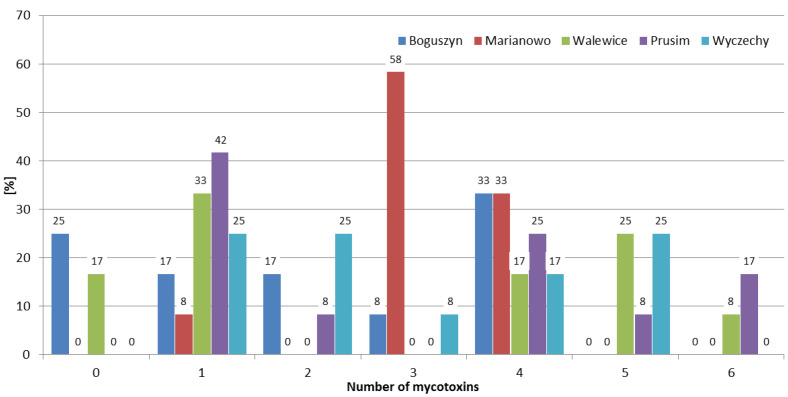
Distribution of the number of mycotoxins in different samples collection location.

Although mycotoxins produced by *Fusarium* molds are a major concern worldwide, there are few published articles regarding the interaction between them. Most of the research shows synergistic or additive effects on the different parameters of animals’ well-being [27]. Therefore, it is extremely important to determine not just one, but the largest possible number of mycotoxins in the samples tested. In the 2016/2017 growing season, at least one mycotoxin was detected in all samples, and 70% of the samples contained four or more mycotoxins. While in the 2018/2019 growing season, 70% of the samples contained one or no mycotoxins, and four or more mycotoxins were found in only 10% of the rye samples (Figure 3). The frequencies of mycotoxin occurrence in different rye varieties were similar (Figure 4). In Marianowo, three or more mycotoxins were present in 92% of the samples, while in other locations these values were around 50% (Figure 5).

In earlier research in Poland, Błajet-Kosicka et al. [17] found that over 50% of the rye samples contained at least two mycotoxins, and over 5% of the samples contained five or more mycotoxins. In Lithuania, Mankevičienė et al. [18] analyzed DON, ZEN, and T-2 toxin in winter cereals in 2006 and 2007, and found that two or more of these mycotoxins were present in 84% and 96% of the samples, respectively. In the presented study, two or more mycotoxins were detected in 67% of the samples; the results are lower than those reported by Mankevičienė et al. [18], while higher than those presented by Błajet-Kosicka et al. [17].

**Figure 6:**
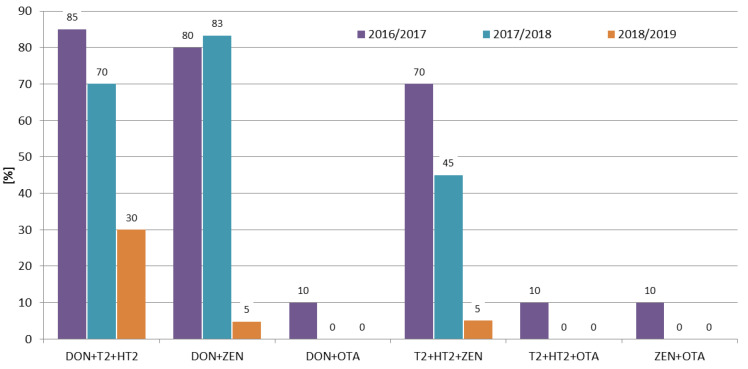
Percentage of co-occurrence of mycotoxins in different growing seasons.

**Figure 7:**
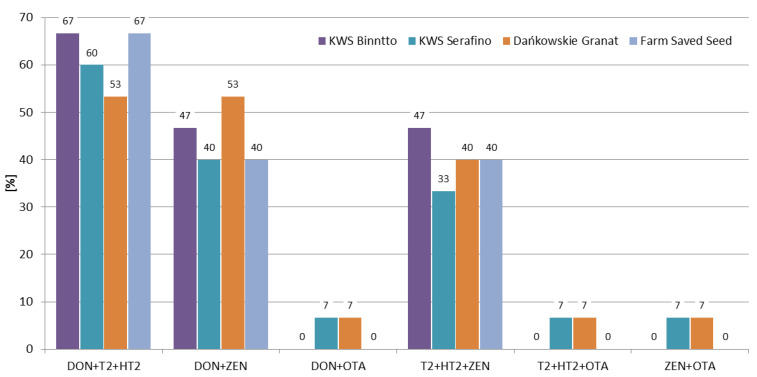
Percentage of co-occurrence of mycotoxins in different rye varieties.

**Figure 8:**
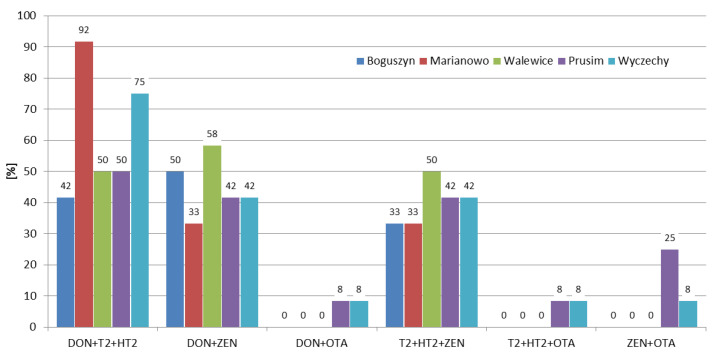
Percentage of co-occurrence of mycotoxins in different locations.

The combinations between mycotoxins were calculated for the most frequently occurring ones (DON, ZEN, T-2 toxin, and HT-2 toxin), as well as for ochratoxin A (since it is regulated by the EU). In the 2016/2017 and 2017/2018 growing seasons the most frequently co-occurring combinations were DON + ZEN and DON + T-2 + HT-2. In the 2018/2019 growing season the combination of DON + T-2 + HT-2 was the most frequent one (30% of the samples) (Figure 6). Considering the variety of rye and the location, the co-occurrence rates were similar for a given combination (Figure 7, Figure 8).

In recent years, several researchers have presented the co-occurrence of the most important mycotoxins in rye samples. In our previous study, Błajet-Kosicka et al. [17], we examined 76 rye grain samples and found that combinations of DON + ZEN (15.4% of all samples) and DON + T-2 + HT-2 + ZEN (14.5% of the samples), were the most common. In Lithuania, Mankevičienė et al. [18] analyzed *Fusarium* mycotoxins in 32 (in 2006) and 28 (in 2007) winter grain samples (including rye), and found that combinations of DON + ZEN (13% of the samples) and DON + T-2 + ZEN (70% of the samples) were present at the highest percentage in 2006 and 2007, respectively. Tutelyan et al. [19] found T-2 + HT-2 in 13% of 23 rye samples from Russia, and the co-occurrence of T-2 + HT-2 + ZEN and ZEN + HT-2 was found in 4% of the samples. In the present study, the co-occurrence of DON + ZEN (45% of all samples) was higher than the results reported by Błajet-Kosicka et al. [17] and Mankevičienė et al. [18].

Table 4 shows the correlations between individual mycotoxin levels in the analyzed rye samples. Due to the low contamination rate of most mycotoxins, we determined correlations for the most frequently occurring ones, i.e., DON, T-2, HT-2, and ZEN. A highly significant (*p* < 0.001) correlation was determined for T-2 and HT-2 (r = 0.840), as well as for DON and ZEN (r = 0.708). In contrast, the correlations between DON and T-2, DON and HT-2, T-2 and ZEN, and HT-2 and ZEN were not significant (*p* = 0.528, *p* = 0.816, *p* = 0.745, and *p* = 0.930, respectively). The fact that DON and ZEN as well as T-2 and HT-2 can be produced by the same molds can explain their correlations [4]. In previous research in Poland, Błajet-Kosicka et al. [17] analyzed 76 rye samples and observed statistically confirmed dependencies between DON and ZEN, T-2 and HT-2, as well as T-2 and ZEN (*r* = 0.579, *r* = 0.586, and *r* = 0.556, respectively). In Germany, Gottschalk et al. [21] determined a correlation between T-2 and HT-2, as well as DON and 15acetylDON (*r* = 0.873 and *r* = 0.957), in 61 rye grain samples, but due to the low concentrations of these toxins, the correlations were less evident than in other analyzed grains (wheat and oats). In Lithuania, Mankevičienė et al. [28] observed statistically significant correlations between DON and ZEN, DON and T-2, as well as T-2 and ZEN in winter grains (*r* = 0.624, *r* = 0.717, and *r* = 0.834, respectively); however, they were dependent on the year of research and the variety. The determined correlations in the presented study are in agreement with the results reported by Błajet-Kosicka et al. [17], Gottschalk et al. [21], and Mankevičienė et al. [28].

## 3. Conclusions

The occurrence of *Fusarium* mycotoxins (DON, NIV, 3ADON, MAS, DAS, T-2, HT-2, and ZEN), as well as OTA in 60 winter rye samples of four varieties (KWS Binntto, KWS Serafino, Dańkowskie Granat, and Farm Saved Seed) cultivated in three consecutive growing seasons in five different regions of Poland was determined using LC-MS/MS and LC-FLD. DON was determined in 90% of the samples, followed by T-2, HT-2, and ZEN (63%, 57%, and 45% positive results, respectively). The mean concentrations of these four analytes were: 28.8 µg/kg (maximum 354.1 µg/kg), 0.98 µg/kg (maximum 6.63 µg/kg), 2.98 µg/kg (maximum 29.8 µg/kg), and 0.69 µg/kg (maximum 10.2 µg/kg), respectively. OTA, a storage mycotoxin, was present in 3% of the samples, with a maximum concentration of 2.75 µg/kg. The mean concentrations for individual mycotoxins were highest in the 2016/2017 growing season, e.g., for DON 70.6 µg/kg, 9.62 µg/kg, and 6.21 µg/kg, respectively, in three consecutive vegetation seasons; for T-2 1.64 µg/kg, 0.96 µg/kg, and 0.36 µg/kg, respectively. Weather conditions (the lowest mean temperature and the highest total precipitation) in the first year of study were favorable for development of *Fusarium* fungi compared to the other two growing seasons. In the 2016/2017 growing season, 95% of the samples contained at least two mycotoxins and 70% of the samples contained four or more mycotoxins, while in the 2018/2019 growing season, 70% of the samples contained one or no mycotoxins, and four or more mycotoxins were found in only 10% of the rye samples. The frequencies of the mycotoxins’ occurrence in different rye varieties were similar. In Marianowo three or more mycotoxins were present in 92% of the samples, while in other locations these values were around 50%. None of the analyzed rye samples exceeded the maximum acceptable mycotoxin level set by the European Commission. Although mycotoxins are one of the most important problems in crop and animal production worldwide, little is known so far about the possible existence of synergistic or additive effects regarding the interaction between them, making it difficult to assess the impact of such effects on the different parameters of animals’ well-being. For this reason, it is very important to determine not only individual mycotoxins, but as many of them as possible in grain samples, which in the future may lead to a better understanding of the effects of combinations of mycotoxins on animal health.

## 4. Materials and Methods 

### 4.1. Samples

**Figure 9:**
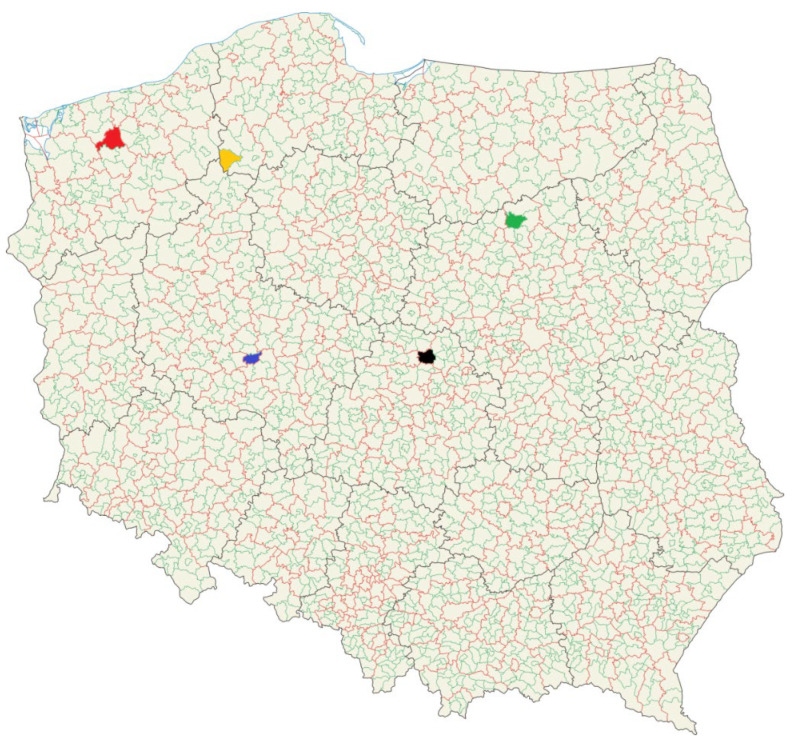
Five locations in Poland where rye samples were cultivated and collected (Boguszyn–blue; Prusim–red; Walewice–yellow; Marianowo–green; Wyczechy–black).

Four winter rye varieties, KWS Binntto (hybrid), KWS Serafino (hybrid), Dańkowskie Granat (population), and farm saved seed (FSS)—non certified seeds multiplied on a farm over five times (population)—were collected from trial fields in five regions of Poland (Figure 9). Field experiments were conducted in three consecutive seasons (2017–2019) using a randomized complete block method with four replications and a plot size of 10 m^2^. Rye was sown before the end of September in soil classes IVa, IVb, and V. The weather conditions (mean temperature and precipitation) during the growing seasons were collected from the Institute of Meteorology and Water Management [29]. Nitrogen fertilization was applied at levels of 100–140 kg N/ha, depending on the quality and richness of the soil. Herbicides and insecticides were used at appropriate frequencies and doses to provide adequate yields. Rye samples (500g per sample) were ground using a Retsch (Haan, Germany) ZM 200 laboratory mill, and stored in a cold, dry location until analysis.

### 4.2. Chemicals

The mycotoxins DON, NIV, 3ADON, MAS, DAS, T2, HT2, ZEN, and OTA, as well as the internal standards 13C-DON, 13C-T2, 13C-HT2, and 13C-ZEN were bought from Romer Labs (Tulln, Austria). Acetonitrile (gradient grade), methanol (LC-MS grade), ammonium acetate (LC-MS grade), and acetic acid (LC-MS grade) were purchased from Merck (Darmstadt, Germany). Water for analysis was purified using Elix 3 and Simplicity UV systems from Merck (Darmstadt, Germany).

### 4.3. Sample Preparation

#### 4.3.1. Trichothecenes and Zearalenone

Ground cereal grain samples (12.5 g) were extracted for 3 min using a homogenizer (Heidolph, Schwabach, Germany) with 50 mL acetonitrile/water (8/2, *v*/*v*). The extracts were then filtered with fluted filter paper (Vicam, Germany). 40 µL of 13C-ZEN (c = 1.00 µg/mL) was added to 4 mL of the extract, and after vortexing the mixture was applied to a BondElute Mycotoxin column (Agilent, Santa Clara, CA, USA). Afterwards, 2 mL of the purified extracts were mixed with 50 µL of the internal standard solution (13C-DON c = 2.50 µg/mL, 13C-T2 c = 0.25 µg/mL, and 13C-HT2 c = 0.25 µg/mL) and evaporated to dryness at 50 °C under a stream of nitrogen. The residues were reconstituted in 0.495 mL of methanol/water mixture (2/8, *v*/*v*) by vortexing for 1 min.

Trichothecenes and zearalenone were determined using high-performance liquid chromatography (HPLC) with tandem mass spectrometry MS/MS detection. The HPLC analyses were performed using a Shimadzu Nexera instrument with a Gemini-NX-C18 chromatographic column (150 × 4.6 mm, 3 µm) from Phenomenex (Torrance, USA), at 25 °C using gradient elution. Both Mobile Phase A—1% acetic acid in water—and B—1% acetic acid in methanol—contained 5 mM ammonium acetate. Elution with 70% of Mobile Phase A was maintained for 0.5 min; subsequently a linear gradient was applied, reaching 90% of Mobile Phase B after 5.5 min, being run isocratically for 4 min, and then being taken back to 70% of the Mobile Phase A, which was maintained till the end of the run at 15.0 min. The flow rate was set to 0.5 mL/min, and the injection volume was 7 µL. Mass spectrometry was performed using an API 4000 (Sciex, Framingham, MA, USA). The electrospray ionization (ESI) interface was used in negative and positive ion modes at 500 °C with the following settings: curtain gas 25 psi, nebulizer gas 50 psi, auxiliary gas 50 psi, collision gas 6, ionization voltage −4000 or +4500 V. Optimized analyte-dependent MS/MS parameters are given in Table 5.

The concentrations of mycotoxins were calculated using external calibration. The limits of detection (LOD) and limits of quantitation (LOQ) obtained for each mycotoxin using the analytical method are presented in Table 6. The LOD (signal-to-noise ratio of 3) and LOQ (signal-to-noise ratio of 10), respectively, were estimated using Analyst 1.6.2 software (Sciex, Framingham, USA) by spiking blank extract before extraction with low concentrations of analytes.

#### 4.3.2. Ochratoxin A

Ground cereal grain samples (12.5 g) were homogenized for 2 min with 50 mL of acetonitrile/water (6:4, *v*/*v*). The extracts were filtered with fluted filter paper (Vicam, Germany), and 5 mL aliquots were added to 55 mL of phosphate-buffered saline solution, and the mixtures were filtered again using microfiber filters (Vicam, Germany). Subsequently, 48 mL of the diluted extracts were applied to Ochraprep columns (R-Biopharm Rhone Ltd., Glasgow, UK). The columns were washed with 20 mL of water and dried with air. OTA was eluted using 1.5 mL of methanol/acetic acid (98/2, *v*/*v*). The eluates were collected in 4 mL sample vials, 1.5 mL of water was passed through the columns, and the samples were vortexed.

Ochratoxin A was determined using HPLC with fluorescence detection. An HPLC system (Merck-Hitachi, Darmstadt, Germany) comprising an L-2130 pump, L-2200 autosampler, L-2300 column oven, and L-2480 fluorescence detector was used. Chromatographic separation was achieved on a LiChrospher 100 RP-18 column (250 × 4.0 mm, 5 μm) from Merck (Darmstadt, Germany), equipped with a C-18 guard column, at 25 °C using isocratic elution. The mobile phase consisted of acetonitrile/2% acetic acid (7:3, *v*/*v*). The flow rate was 1 mL/min, and the injection volume was 50 μL. The excitation and emission wavelengths of the fluorescence detector were set at 330 nm and 460 nm, respectively. The data was processed using EZChrom Elite version 3.1.1 software. The limits of detection and quantification were 0.13 µg/kg and 0.40 µg/kg, respectively, while the recovery value was 96%.

### 4.4. Statistics

Descriptive statistics, including number of samples, percentage of positive results, mean, mean of positive samples, median and maximum concentrations, as well as Pearson’s correlation coefficient and statistical significance (for a significance level α = 0.001), were calculated using MS Excel 2013 (Microsoft). A one-way analysis of variance (ANOVA) and Tukey’s test at α = 0.05 were used to assess the significance of the differences between mycotoxin concentrations (Statistica 13, StatSoft, Poland). For the calculation, the results below the LOD value were set as zero, whereas the results between the LOD and the LOQ were set as the LOQ value for each mycotoxin.

## Figures and Tables

**Table 1:** Concentrations of mycotoxins in investigated rye samples in different growing seasons.

	DON	NIV	3ADON	MAS	DAS	T2	HT2	ZEN	OTA
Growing Season 2016/2017									
Number of samples	20	20	20	20	20	20	20	20	20
Positive (%)	100	15	10	5	0	85	85	80	10
Mean (µg/kg)	70.6 ^c^	<LOQ	<LOD	<LOD	<LOD	1.64 ^c^	6.59 ^b^	1.82 ^b^	<LOQ
Mean positive (µg/kg)	70.6	8.06	<LOQ	<LOQ	<LOD	1.93	7.75	2.27	1.58
Median (µg/kg)	45.2	<LOD	<LOD	<LOD	<LOD	0.98	3.95	0.60	<LOD
Maximum (µg/kg)	354.1	9.72	3.00	<LOQ	<LOD	6.63	29.8	10.2	2.75
**Growing Season 2017/2018**									
Number of samples	20	20	20	20	20	20	20	20	20
Positive (%)	90	10	0	10	0	70	55	50	0
Mean (µg/kg)	9.62 ^b^	<LOQ	<LOD	<LOD	<LOD	0.96 ^b^	<LOQ ^a^	0.24 ^a^	<LOD
Mean positive (µg/kg)	10.7	15.5	<LOD	2.29	<LOD	1.37	2.27	0.49	<LOD
Median (µg/kg)	8.44	<LOD	<LOD	<LOD	<LOD	0.61	<LOQ	<LOQ	<LOD
Maximum (µg/kg)	27.9	26.4	<LOD	3.07	<LOD	5.62	3.25	1.99	<LOD
**Growing Season 2018/2019**									
Number of samples	20	20	20	20	20	20	20	20	20
Positive (%)	80	5	0	5	0	35	30	5	0
Mean (µg/kg)	6.21 ^a^	<LOD	<LOD	<LOD	<LOD	<LOQ ^a^	<LOQ ^a^	<LOD	<LOD
Mean positive (µg/kg)	7.77	<LOQ	<LOD	<LOQ	<LOD	1.03	3.64	<LOQ	<LOD
Median (µg/kg)	4.17	<LOD	<LOD	<LOD	<LOD	<LOD	<LOD	<LOD	<LOD
Maximum (µg/kg)	17.0	<LOQ	<LOD	<LOQ	<LOD	1.68	6.93	<LOQ	<LOD

For the calculation, the results below the limit of detection (LOD) value were set as zero, whereas the results comprising between the LOD and the limit of quantification (LOQ) were set as the LOQ value for each mycotoxin. Mean concentrations in the same column with different letters a–c are significantly different (α = 0.05).

**Table 2:** Concentrations of mycotoxins in investigated rye samples in different samples collection location.

	DON	NIV	3ADON	MAS	DAS	T2	HT2	ZEN	OTA
Boguszyn									
Number of samples	12	12	12	12	12	12	12	12	12
Positive (%)	75	0	0	0	0	42	42	50	0
Mean (µg/kg)	13.3 ^a^	<LOD	<LOD	<LOD	<LOD	<LOQ ^a^	<LOQ ^a^	0.28 ^b^	<LOD
Mean positive (µg/kg)	17.8	<LOD	<LOD	<LOD	<LOD	0.91	2.12	0.55	<LOD
Median (µg/kg)	10.2	<LOD	<LOD	<LOD	<LOD	<LOD	<LOD	<LOQ	<LOD
Maximum (µg/kg)	42.2	<LOD	<LOD	<LOD	<LOD	1.22	2.58	1.92	<LOD
**Marianowo**									
Number of samples	12	12	12	12	12	12	12	12	12
Positive (%)	100	0	0	0	0	92	92	33	0
Mean (µg/kg)	13.5 ^a^	<LOD	<LOD	<LOD	<LOD	2.03 ^d^	7.43 ^c^	<LOQ ^a^	<LOD
Mean positive (µg/kg)	13.5	<LOD	<LOD	<LOD	<LOD	2.21	8.10	0.23	<LOD
Median (µg/kg)	8.20	<LOD	<LOD	<LOD	<LOD	1.13	2.70	<LOD	<LOD
Maximum (µg/kg)	48.2	<LOD	<LOD	<LOD	<LOD	6.63	29.8	0.30	<LOD
**Walewice**									
Number of samples	12	12	12	12	12	12	12	12	12
Positive (%)	100	17	0	25	0	50	50	58	0
Mean (µg/kg)	19.3 ^b^	<LOQ	<LOD	<LOQ	<LOD	0.98 ^b^	3.38 ^b^	0.37 ^c^	<LOD
Mean positive (µg/kg)	19.3	15.5	<LOD	2.02	<LOD	1.96	6.77	0.63	<LOD
Median (µg/kg)	14.0	<LOD	<LOD	<LOD	<LOD	<LOQ	<LOQ	0.21	<LOD
Maximum (µg/kg)	48.3	26.4	<LOD	3.07	<LOD	3.01	14.47	1.99	<LOD
**Prusim**									
Number of samples	12	12	12	12	12	12	12	12	12
Positive (%)	75	25	8	8	0	58	50	42	8
Mean (µg/kg)	44.0 ^c^	<LOQ	<LOD	<LOD	<LOD	<LOQ ^a^	<LOQ ^a^	0.93 ^d^	<LOQ
Mean positive (µg/kg)	58.6	6.10	<LOQ	<LOQ	<LOD	0.74	3.58	2.24	2.75
Median (µg/kg)	<LOQ	<LOD	<LOD	<LOD	<LOD	<LOQ	<LOQ	<LOD	<LOD
Maximum (µg/kg)	210.7	9.72	<LOQ	<LOQ	<LOD	1.44	5.51	3.82	2.75
**Wyczechy**									
Number of samples	12	12	12	12	12	12	12	12	12
Positive (%)	100	8	8	0	0	75	50	42	8
Mean (µg/kg)	54.1 ^d^	<LOD	<LOD	<LOD	<LOD	1.10 ^c^	<LOQ ^a^	1.80 ^e^	<LOD
Mean positive (µg/kg)	54.1	8.89	<LOQ	<LOD	<LOD	1.47	2.80	4.32	<LOQ
Median (µg/kg)	12.9	<LOD	<LOD	<LOD	<LOD	<LOQ	<LOQ	<LOD	<LOD
Maximum (µg/kg)	354.1	8.89	<LOQ	<LOD	<LOD	5.62	3.57	10.2	<LOQ

For the calculation, the results below the LOD value were set as zero, whereas the results comprising between the LOD and the LOQ were set as the LOQ value for each mycotoxin. Mean concentrations in the same column with different letters a-e are significantly different (α = 0.05).

**Table 3:** Concentrations of mycotoxins in investigated rye samples in different varieties.

	DON	NIV	3ADON	MAS	DAS	T2	HT2	ZEN	OTA
KWS Binntto hybrid rye									
Number of samples	15	15	15	15	15	15	15	15	15
Positive (%)	87	20	7	7	0	67	60	47	0
Mean (µg/kg)	24.1 ^b^	<LOQ	<LOD	<LOD	<LOD	1.06 ^c^	4.01 ^c^	1.13 ^d^	<LOD
Mean positive (µg/kg)	27.8	15.0	<LOQ	3.07	<LOD	1.59	6.69	2.42	<LOD
Median (µg/kg)	12.1	<LOD	<LOD	<LOD	<LOD	<LOQ	<LOQ	<LOD	<LOD
Maximum (µg/kg)	136.7	26.4	<LOQ	3.07	<LOD	5.42	23.9	10.2	<LOD
**KWS Serafino Hybrid Rye**									
Number of samples	15	15	15	15	15	15	15	15	15
Positive (%)	93	7	0	7	0	60	53	40	7
Mean (µg/kg)	28.4 ^c^	<LOD	<LOD	<LOD	<LOD	0.80 ^a^	2.03 ^b^	0.35 ^a^	<LOQ
Mean positive (µg/kg)	30.4	4.65	<LOD	<LOQ	<LOD	1.34	3.81	0.87	2.75
Median (µg/kg)	9.05	<LOD	<LOD	<LOD	<LOD	0.65	<LOQ	<LOD	<LOD
Maximum (µg/kg)	210.7	4.65	<LOD	<LOQ	<LOD	3.00	9.88	3.28	2.75
**Dańkowskie Granat Population Rye**									
Number of samples	15	15	15	15	15	15	15	15	15
Positive (%)	87	7	0	7	0	60	53	53	7
Mean (µg/kg)	19.3 ^a^	<LOD	<LOD	<LOD	<LOD	0.86 ^b^	<LOQ ^a^	0.59 ^b^	<LOD
Mean positive (µg/kg)	22.3	5.58	<LOD	<LOQ	<LOD	1.43	3.42	1.10	<LOQ
Median (µg/kg)	7.83	<LOD	<LOD	<LOD	<LOD	<LOQ	<LOQ	<LOQ	<LOD
Maximum (µg/kg)	96.6	5.58	<LOD	<LOQ	<LOD	5.62	6.51	3.64	<LOQ
**Farm Saved Seed Population Rye**									
Number of samples	15	15	15	15	15	15	15	15	15
Positive (%)	93	7	7	7	0	67	60	40	0
Mean (µg/kg)	43.5 ^d^	<LOD	<LOD	<LOD	<LOD	1.21 ^d^	4.04 ^c^	0.70 ^c^	<LOD
Mean positive (µg/kg)	46.7	<LOQ	<LOQ	<LOQ	<LOD	1.82	6.73	1.75	<LOD
Median (µg/kg)	11.3	<LOD	<LOD	<LOD	<LOD	<LOQ	<LOQ	<LOD	<LOD
Maximum (µg/kg)	354.1	<LOQ	<LOQ	<LOQ	<LOD	6.63	29.76	6.79	<LOD

For the calculation, the results below the LOD value were set as zero, whereas the results comprising between the LOD and the LOQ were set as the LOQ value for each mycotoxin. Mean concentrations in the same column with different letters a-d are significantly different (α = 0.05).

**Table 4:** Correlation between individual mycotoxins in rye samples.

	DON	T2	HT2	ZEN
DON	1			
T2	0.124	1		
HT2	0.134	0.840 ^a^	1	
ZEN	0.708 ^a^	0.001	0.010	1

^a^ Significant at *p* < 0.001.

**Table 5:** Optimized electrospray ionization tandem mass spectrometry (ESI)-MS/MS conditions for analytical method.

		Precursor Ion [m/z]	Product Ions [*m*/*z*] *	Declustering Potential [V]	Collision Energy [V]	Cell Exit Potential [V]
NIV	[M+Ac]^−^	371.1	281.0/59.0	−40	−22/−40	−14/−5
DON	[M+Ac]^−^	355.1	264.8/58.9	−35	−20/−38	−17/−1
13C-DON	[M+Ac]^−^	370.2	310.0	−50	−14	−7
3ADON	[M+Ac]^−^	397.1	307.0/59.0	−50	−18/−42	−7/−1
MAS	[M+NH_4_]^+^	342.1	265.0/107.1	46	13/19	18/6
DAS	[M+NH_4_]^+^	384.1	307.0/247.0	51	17/19	20/16
HT2	[M+NH_4_]^+^	442.2	215.0/263.0	51	19/17	14/18
13C-HT2	[M+NH_4_]^+^	464.1	278.1	51	17	18
T2	[M+NH_4_]^+^	484.1	215.0/185.0	61	25/29	14/12
13C-T2	[M+NH_4_]^+^	508.3	322.1	61	19	8
ZEN	[M-H]^−^	317.1	130.8/174.9	−85	−40/−32	−7/−9
13C-ZEN	[M-H]^−^	335.1	139.9	−100	−42	−7

* Values are given in the order quantifier/qualifier.

**Table 6:** Method performance parameters.

Mycotoxin	LOD (µg/kg)	LOQ (µg/kg)	Linearity (µg/kg)	Recovery (%) ± RSD (*n* = 3)
NIV	1.0	3.0	5–1000	75 ± 4
DON	1.0	3.0	5–2000	90 ± 2
3ADON	1.0	3.0	5–500	90 ± 7
MAS	0.50	1.5	5–500	85 ± 5
DAS	0.33	1.0	5–500	90 ± 3
HT2	0.67	2.0	5–1000	82 ± 4
T2	0.20	0.60	5–1000	88 ± 3
ZEN	0.07	0.20	5–500	110 ± 5

## Abbreviations

3ADON	3-acetyl-deoxynivalenol
DAS	diacetoxyscirpenol
DON	deoxynivalenol
FHB	*Fusarium* head blight
HT-2	HT-2 toxin
LOD	limit of detection
LOQ	limit of quantification
MAS	monoacetoxuscirpenol
NIV	nivalenol
OTA	ochratoxin A
T2	T-2 toxin
ZEN	zearalenone
